# Transcutaneous Puncture of an Undeflatable Coronary Angioplasty Balloon Catheter

**DOI:** 10.1155/2018/6252809

**Published:** 2018-09-03

**Authors:** Gregor Leibundgut, Christian Degen, Florian Riede

**Affiliations:** ^1^Department of Cardiology, Medizinische Universitätsklinik, Kantonsspital Baselland, Rheinstrasse 26, 4410 Liestal, Switzerland; ^2^Departement of Internal Medicine, Medizinische Universitätsklinik, Kantonsspital Baselland, Rheinstrasse 26, 4410 Liestal, Switzerland

## Abstract

This case report describes a quick and safe method to successfully retrieve an undeflatable angioplasty balloon via the transradial access site. The article also presents potential mechanisms of contrast media entrapment and bench tests of guidewire-assisted balloon puncture. After successful stent implantation in the proximal right coronary artery in a 74-year-old female patient referred for acute coronary syndrome, the balloon catheter became undeflatable for an unknown reason. Several attempts to burst the balloon in the guide catheter and the aorta failed. After a pullback into the radial artery, the still inflated balloon became stuck again and was unable to be retrieved through the sheath. Finally, transcutaneous puncture and aspiration of contrast media with a 23 gauge syringe needle through the skin in the right forearm allowed deflation and successful removal the balloon.

## 1. Introduction

Transradial access for percutaneous coronary interventions (PCI) has become more frequent in recent years [1]. In particular, patients presenting with ST-segment elevation myocardial infarction (STEMI), non-STEMI (NSTEMI), and cardiogenic shock benefit from the radial approach [2–8]. Based on these studies, the latest ESC guidelines recommend the transradial access for the management of acute coronary syndromes (ACS) (class I, level A) [9]. Radial access is also associated with reduced incidence of acute kidney injury after PCI [10].

Optimized guide support, the latest guidewire and balloon technologies, and additional accessories such as extension catheters provide enough backup to successfully cross severely calcified lesions via the radial route. However, more complex procedures inevitably result in more complications. Smaller-sized guide catheters with the radial approach may limit the ability to remove damaged gear through the access site. Undeployed or lost stents, broken guidewires, twisted guiding catheters, trapped balloon catheters, and other interventional tools have been successfully removed by the femoral access or cardiac surgery [11]. Only a few reports describing true transradial bailout methods have been published [12]. The use of large balloon diameters and lengths concurrently with smaller guiding catheters may further increase complication rates.

This case report describes an easy method for successful transradial retrieval of an undeflatable 3.5/48 balloon catheter using transcutaneous puncture and contrast aspiration in the forearm.

## 2. Case Report

**Figure 1:**
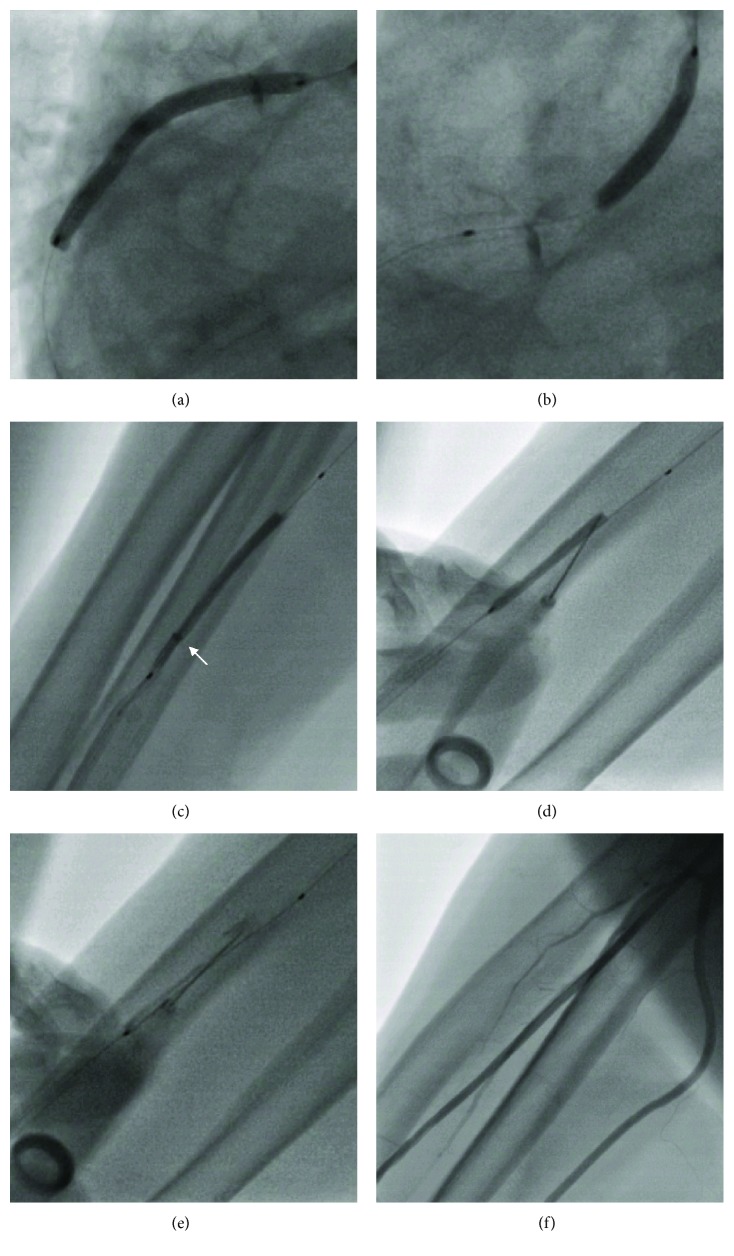
Angiography. (a) Implantation of XIENCE Xpedition 3.5/48 DES. (b) Undeflatable balloon stuck to the RCA ostium. (c) Undeflated balloon in the radial artery. (d) Transcutaneous puncture with a 23 gauge needle. (e) Aspiration of contrast media with complete deflation of the balloon. (f) Injection of contrast media through the radial sheath confirms patent radial artery.

A 74-year-old female patient with an acute non-ST-segment elevation myocardial infarction (NSTEMI) was referred for coronary angiography. Transradial access was established with a 10 cm 6F Introducer sheath (Glidesheath, Terumo Medical Corporation). Coronary angiography with a 6F Judkins right 4 diagnostic catheter revealed a long and heavily calcified lesion of the proximal segment and ostium of the right coronary artery (RCA). During the retrieval of the diagnostic catheter, the patient complained of severe pain in her right arm due to spasm of the radial artery. After repetitive intravenous administration of midazolam up to 8 mg and nitroglycerine through the radial sheath, retrieval of the catheter was finally possible. PCI of the lesion in the proximal RCA was then performed over a 5F Amplatz left 1 guiding catheter (Launcher, Medtronic) using an extra support guidewire (Galeo ES, Biotronik) and a 3.0/15 semi compliant balloon (Across HP, Acrostak) for predilatation. A 3.5/48 drug-eluting stent (DES) (XIENCE Xpedition, Abbot Vascular) was then placed in the predilated coronary segment and inflated to 18 atm (Figure 1(a)). A mixture of contrast media and saline at a ratio of 1 : 1 was used for all balloon inflations. After a second more proximal inflation, the balloon catheter did not deflate completely and the operator was unable to pull it back into the 5F guiding catheter (Figure 1(b)). Excessive inflation and deflation with the indeflator and forced aspiration with a 50 ml syringe were unable to deflate the balloon that was stuck at the RCA ostium. The operator then decided to pull back the entire equipment including the guiding catheter, guidewire, and stent balloon at once. Finally, the balloon catheter became released from the stent remaining in place in the coronary artery. An attempt to burst the balloon in the ascending aorta by inflation well over rated burst pressure (RBP), as well as puncturing the balloon within the guide catheter with multiple stiff guidewires, also failed.

During a further pullback of the system, the still inflated balloon became stuck again in the small and vasospastic radial artery. The operator was unable to pull or push the balloon in any direction and was unable to wedge the balloon into the 6F radial sheath. Moreover, a forced pullback of the partially inflated balloon catheter resulted in invagination of the distal part of the balloon (Figure 1(c), white arrow). After injecting local anesthesia on the middle forearm, transcutaneous puncture of the balloon through the skin and the radial artery wall was performed with a 23 gauge syringe needle using fluoroscopy for guidance (Figure 1(d)), and contrast media were aspirated with the syringe (Figure 1(e)). Retrieval of the deflated balloon was then possible through the radial sheath. Reintubation of the RCA with a fresh guiding catheter revealed a good angiographic result in the proximal segment and relevant acute recoil at the ostium of the calcified RCA. Implantation of a second short 4.0/9 mm DES (BMX-Alpha, Biosensors) was necessary to mechanically stabilize the lesion. Injection of contrast media through the sheath confirmed the integrity of the radial artery at the puncture site (Figure 1(f)). Radial compression was used to close the 6F access site. The clinical follow-up remained uneventful.

## 3. Discussion

The presented case describes a quick and safe method to successfully retrieve an undeflatable angioplasty balloon via the transradial access site. Transcutaneous puncture can easily be performed by palpation of the balloon or using fluoroscopy for guidance. Puncture of the balloon catheter alone may be sufficient for retrieval through the sheath; however, aspiration of contrast media with the syringe facilitates balloon deflation and may be beneficial.

**Figure 2:**
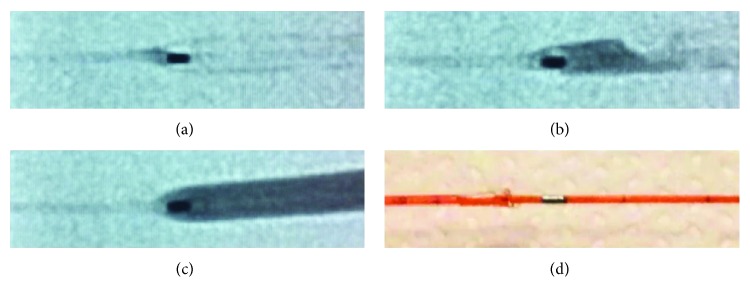
Exit/Entrance port of contrast media. (a), (b), and (c) show entrance of contrast media filling the angioplasty balloon by X-ray. (d) Photograph of a balloon catheter with the balloon cut from the rest of the catheter.

**Figure 3:**
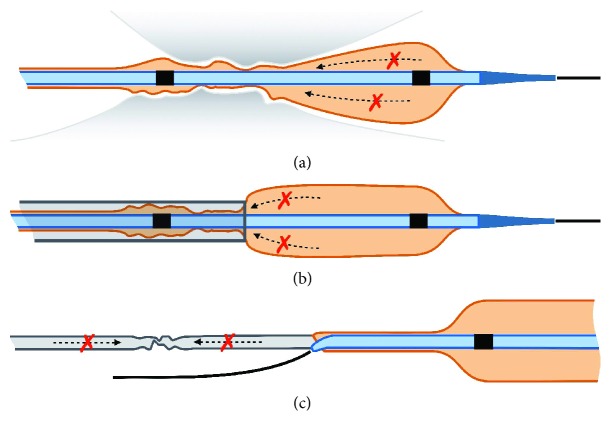
Mechanisms of contrast media entrapment. Several potential mechanisms can result in the contrast media being trapped inside the balloon. (a) Acute recoil of calcified lesion or stent and (b) strangulation of the balloon in the guide catheter. (c) Damage to the hypotube (kink or break). Dotted arrows and red X indicate compromised contrast media flow from the balloon into the hypotube.

Contrast media exit the balloon at the proximal end (Figure 2) and flows through the flexible distal part of the catheter shaft and then through the metallic hypotube. Viscosity plays an important role with deflation time depending on the mixing ratio between contrast media and saline solution [13]. Potential mechanisms of contrast media entrapment in angioplasty balloons are illustrated in Figure 3 and include (A) acute recoil of a severely calcified lesion after balloon inflation with compression of the deflating balloon around the entry port; (B) strangulation of the proximal end of the balloon by the guiding catheter after the early pullback of the balloon catheter before complete deflation; and (C) damage to the hypotube. The final common scenario is that the balloon can only be partially deflated due to the entrapment of contrast media, which further complicates retrieval of the balloon catheter. In the first two situations, contrast media collect in the distal part of the balloon and this can complicate balloon retrieval. Only the third mechanism explains all features observed in the current case including distal deflation of the balloon and inability to inflate the balloon again. In the present case, however, a collection of contrast media in the proximal part could also have been a result from incomplete deaeration prior to initial inflation. The heavily calcified ostium and the acute recoil after stent implantation were very likely the reason for the balloon entrapment. Longitudinal stent compression (LSC) is frequently found in ostial lesions and may further complicate acute recoil and removal of the distally placed gear [14]. However, no angiographic characteristics of LSC were found in the present case.

A classic bailout method for undeflatable angioplasty balloons includes prolonged/forced balloon inflation well above rated burst pressures (RBP) to intentionally burst the balloon. This maneuver should be performed in a large vessel such as the ascending aorta or subclavian artery to avoid rupture of the coronary artery. This can be difficult with noncompliant (NC) and other high and very high-pressure balloons such as the OPN NC (SIS Medical, Switzerland) that can withstand up to 50 atm. The fact that inflation was also impossible suggests that the hypotube was obstructed or damaged and no contrast was able to flow in or out of the balloon. However, a close examination of the balloon catheter did not reveal a definite mechanism of failure in this case.

**Figure 4:**
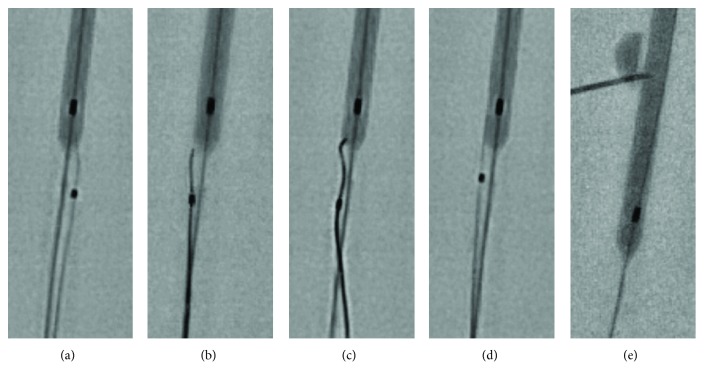
Transluminal balloon puncture. Inflated balloon from a XIENCE Xpedition 3.5/48 DES in a 6F guide catheter. Microcatheter placed proximal to the balloon for optimal guidewire support. Serial failed attempts to puncture the balloon with (a) Conquest Pro 12, (b) Stingray guidewire, (c) Gaia 3, and (d) opposite side of a work horse guidewire. (e) Successful balloon puncture with a 23G syringe needle through the guide catheter wall.

Other potential methods such as transluminal puncture of the balloon at its proximal end within the guide catheter with hard wires like the Gaia 3rd, Confianza Pro 12, Stingray guidewire (stiff wire with 0.0035-inch distal taper), or even the back end of a guidewire failed in bench tests (Figure 4). Even increasing the penetration force with the use of an additional microcatheter failed and is therefore not recommended in such cases. Similar to the present case, Watt and associates also were unable to puncture the balloon using a stiff guidewire and instead had to cut the kinked hypotube to passively let the balloon deflate [15]. Finally, successful balloon puncture is only feasible with a pointed and cut 23 gauge syringe needle and does not leave any damage to the radial artery wall.

Balloon catheters becoming undeflatable are extremely rare complications of PCI. A forced pullback of the balloon catheter of an undeflated balloon may cause the outer body of the distal shaft to shrink, forming a seal with the underlying tube of the distal shaft. At this stage, inflation may help to overcome this seal but may remain permanent once the distal shaft has ruptured. Ruptured balloon shafts leave the balloon behind in the coronary artery, and these cases cannot be resolved with this method.

An easy pullback into large sheaths commonly used in femoral access may account for the rare complication reports. In one published case, the operators were able to retrieve the balloon from the coronary artery and pull it into a 6F femoral sheath [16]. The present case, however, describes a true transradial bailout method without additional access. Using a 5F guide catheter may likely have contributed to the damage of the hypotube in this case. The use of sheathless catheters with a larger lumen may facilitate PCI and further reduce these complications with the radial approach.

## 4. Conclusion

Transcutaneous puncture of an undeflatable balloon catheter in the forearm is feasible and safe and may be considered a true/primary transradial retrieval method.

## References

[1] Ratib K., Mamas M. A., Anderson S. G. (2015). Access site practice and procedural outcomes in relation to clinical presentation in 439,947 patients undergoing percutaneous coronary intervention in the United Kingdom. *JACC: Cardiovascular Interventions*.

[2] Jolly S. S., Yusuf S., Cairns J. (2011). Radial versus femoral access for coronary angiography and intervention in patients with acute coronary syndromes (RIVAL): a randomised, parallel group, multicentre trial. *The Lancet*.

[3] Généreux P., Mehran R., Palmerini T. (2011). Radial access in patients with ST-segment elevation myocardial infarction undergoing primary angioplasty in acute myocardial infarction: the HORIZONS-AMI trial. *EuroIntervention*.

[4] Romagnoli E., Biondi-Zoccai G., Sciahbasi A. (2012). Radial versus femoral randomized investigation in ST-segment elevation acute coronary syndrome. *Journal of the American College of Cardiology*.

[5] Mehta S. R., Jolly S. S., Cairns J. (2012). Effects of radial versus femoral artery access in patients with acute coronary syndromes with or without ST-segment elevation. *Journal of the American College of Cardiology*.

[6] Karrowni W., Vyas A., Giacomino B. (2013). Radial versus femoral access for primary percutaneous interventions in ST-segment elevation myocardial infarction patients: a meta-analysis of randomized controlled trials. *JACC: Cardiovascular Interventions*.

[7] Valgimigli M., Gagnor A., Calabró P. (2015). Radial versus femoral access in patients with acute coronary syndromes undergoing invasive management: a randomised multicentre trial. *The Lancet*.

[8] Pancholy S. B., Palamaner Subash Shantha G., Romagnoli E. (2015). Impact of access site choice on outcomes of patients with cardiogenic shock undergoing percutaneous coronary intervention: a systematic review and meta-analysis. *American Heart Journal*.

[9] Roffi M., Patrono C., Collet J. P. (2016). 2015 ESC Guidelines for the management of acute coronary syndromes in patients presenting without persistent ST-segment elevation: task force for the management of acute coronary syndromes in patients presenting without persistent ST-segment elevation of the European Society of Cardiology (ESC). *European Heart Journal*.

[10] Andò G., Cortese B., Russo F. (2017). Acute kidney injury after radial or femoral access for invasive acute coronary syndrome management: AKI-MATRIX. *Journal of the American College of Cardiology*.

[11] Alexiou K., Kappert U., Knaut M., Matschke K., Tugtekin S. M. (2006). Entrapped coronary catheter remnants and stents: must they be surgically removed?. *Texas Heart Institute Journal*.

[12] Leibundgut G., Löffelhardt N., Neumann F.-J. (2014). Percutaneous retrieval of a twisted guide catheter using a longer second radial sheath. *Catheterization and Cardiovascular Interventions*.

[13] Mogabgab O., Patel V. G., Michael T. T. (2014). Impact of contrast agent viscosity on coronary balloon deflation times: bench testing results. *Journal of Interventional Cardiology*.

[14] Leibundgut G., Gick M., Toma A. (2013). Longitudinal compression of the platinum-chromium everolimus-eluting stent during coronary implantation: predisposing mechanical properties, incidence, and predictors in a large patient cohort. *Catheterization and Cardiovascular Interventions*.

[15] Watt J., Khurana A., Ahmed J. M., Purcell I. F. (2015). Simple solution for an undeflatable stent balloon in the left main stem. *JACC: Cardiovascular Interventions*.

[16] Bostan M., Satiroğlu O., Erdoğan T., Durakoğlugil M. E., Uğurlu Y. (2013). A rare complication: undeflatable balloon of the stent. *Interventional Medicine and Applied Science*.

